# Feasibility, Acceptability, and Efficacy of Virtual Reality Training for Older Adults and People With Disabilities: Single-Arm Pre-Post Study

**DOI:** 10.2196/27640

**Published:** 2021-05-04

**Authors:** Pui Hing Chau, Yan Yan Jojo Kwok, Mee Kie Maggie Chan, Ka Yu Daniel Kwan, Kam Lun Wong, Ying Ho Tang, Kan Lung Peter Chau, Sheung Wa Matthew Lau, Yan Yan Yannex Yiu, Mei Yan Fanny Kwong, Wai Ting Thomas Lai, Mun Kit Leung

**Affiliations:** 1 School of Nursing The University of Hong Kong Hong Kong China; 2 Tung Wah Group of Hospitals Hong Kong China; 3 Department of Industrial and Manufacturing Systems Engineering The University of Hong Kong Hong Kong China

**Keywords:** virtual reality, rehabilitation, older adults, people with disabilities, evaluation

## Abstract

**Background:**

Unlike most virtual reality (VR) training programs that are targeted at homogenous populations, a set of VR games for rehabilitation purposes targeted at a heterogeneous group of users was developed. The VR games covered physical training, cognitive training (classification and reality orientation), community-living skills training, and relaxing scenery experiences. Special considerations for local older adults and people with disabilities were made in terms of hardware choice and software design.

**Objective:**

This study aimed to evaluate the feasibility, acceptance, and efficacy of VR training among users with varying abilities.

**Methods:**

A single-arm pretest-posttest evaluation study was conducted. The participants of the evaluation study were encouraged to undergo 30-minute VR training three times a week for 6 weeks. The 30-minute session consisted of 10 minutes of upper-limb motion games, 10 minutes of lower-limb motion games, and 10 minutes of cognitive games/community-living skills training/relaxing scenery experiences, as appropriate. On completion of each session, usage statistics were documented via the built-in VR software, whereas feedback on the experience of the VR games and adverse events was collected via self-reports and staff observations. Feasibility was reflected by usage statistics, and acceptance was reflected by positive feedback. In addition, health outcomes, including upper-limb dexterity, functional mobility, cognitive function, and happiness, were assessed at baseline, as well as 6 weeks and 3 months after baseline. The primary outcomes were upper-limb dexterity and acceptance of playing VR games.

**Results:**

A total of 135 participants with a mean age of 62.7 years (SD 21.5) were recruited from May 2019 to January 2020, and 124 (91.9%) completed at least one follow-up. Additionally, 76.3% (103/135) of the participants could attend at least 70% of the proposed 18 sessions, and 72.5% (1382/1906) of the sessions had a training time of at least 20 minutes. Linear mixed effect models showed statistically significant effects in terms of upper-limb dexterity (small effect) and cognitive function (moderate effect). Among the 135 participants, 88 provided positive comments. Additionally, 10.4% (14/135) reported mild discomfort, such as dizziness, and none reported severe discomfort.

**Conclusions:**

A set of VR training games for rehabilitation could be applied to users with heterogeneous abilities. Our VR games were acceptable to local older adults and those with different disabilities. Benefits in upper-limb dexterity and cognitive function were observed despite partial compliance to the training protocol. Service providers could refer to our experiences when developing VR training systems for their clients.

## Introduction

Virtual reality (VR) has been widely used in many areas, including education, aviation, medicine, and entertainment. For control and navigation in VR games, users are required to have relatively high cognitive and physical levels. In this regard, researchers and service providers are developing and refining various VR games to extend their applications to older adults and people with disabilities or diseases, who usually have longer response times and cannot handle controllers with buttons because of their weak fine-motor skills. With successful modifications, VR training provides these users the opportunity to engage in motivational training with many repetitions, salient stimuli, and challenging tasks [1]. Examples of VR training, particularly those with a rehabilitation purpose, are well documented in different populations, such as adults with stroke [2], community-dwelling older adults [3], people living with dementia and mild cognitive impairment [4], and children with cerebral palsy and Down syndrome [5].

VR games, particularly those related to living skills training and cognitive training, would be more appealing to users if they are based on local elements and culture that are familiar to the users. Hence, in our local setting (Hong Kong), there are various VR training programs targeting different populations, for example, people with intellectual disability [6,7], acquired brain injury or stroke [8-11], schizophrenia [12], dementia [13], high fall risk [14,15], and different geriatric conditions [16,17].

Despite many VR training programs being developed, they mainly target homogenous populations. There is a lack of an integrated VR training program that suits users with various types of conditions or disabilities. Therefore, there is a need to develop a universal VR training program in Hong Kong to serve a heterogeneous group of users comprising older adults and adults with different disabilities or conditions living in different settings. In 2019, one of the leading nongovernment organizations (NGOs) in Hong Kong took the initiative to develop a universal VR system to supplement the rehabilitation of older adults and people with disabilities, both in the residential care setting and the community setting, with a view to enrich the life experience of service clients and improve their training and treatment outcomes. The common rehabilitation goals of this heterogeneous population were upper-limb dexterity and cognitive status, since these are of utmost importance in maintaining activities of daily living for both institutional and community-dwelling populations. While mobility is also important, it was not one of the common goals, since some of the targeted users already lost this ability. Regarding such development, the efficacy of such VR training in improving health outcomes among users has to be scientifically evaluated. Therefore, the objective of this evaluation study was to investigate the feasibility, acceptance, and efficacy of VR training among older adults and people with different disabilities.

## Methods

### Study Design

This was a service evaluation study employing a single-arm pretest-posttest design. Although a randomized controlled trial or a quasiexperimental design was preferred, such designs would have to involve at least 145 participants per group in order to detect a small-to-medium effect. As the service units might not be able to support recruitment and intervention of such a large sample, these designs could not be adopted.

### Participants

The target population included clients of the service units for rehabilitation or the service units for older adults of one NGO in Hong Kong. The inclusion criteria were service clients of the selected units, covering both residential care settings and community settings; age 18 years or above; ability to understand instructions in VR training; and presence of at least one of the following disabilities: functional impairment, mobility limitation, cognitive impairment, intellectual disability, and visual impairment. The exclusion criteria were conditions that restricted participation in VR training and severe discomfort with VR training.

The VR facility was expected to serve at least 130 clients. Depending on the functional status, cognitive status, and possible attrition among users, it was expected that at least half of the users (ie, 65 participants) would be eligible for outcome assessments. This would result in about 80% power and 5% level of significance to detect a small-to-medium effect size (Cohen *d*=0.35) in pre-post difference.

### Interventions

A set of multipurpose immersive VR games was developed for VR training. These programs fit the language, content, and VR scenes specially designed for Hong Kong individuals, which enhanced usage experience. Various elements, including hardware, virtual setting, gaming operation, and game setting, were specially considered during program development to cater to users with different disabilities and functional levels, so as to ensure user friendliness and comfort for users (Table 1).

The components of the VR games included physical training, cognitive training (classification and reality orientation), community-living skills training, and relaxing scenery experiences (Table 2). To date, the types of VR training, as well as training intensity and frequency to achieve desired outcomes, have not been consistent. Benchmarking with other VR programs for rehabilitation [18-21], the participants of the evaluation study were encouraged to undergo a 30-minute VR training three times a week for 6 weeks. The 30-minute session consisted of 10 minutes of upper-limb motion games, 10 minutes of lower-limb motion games, and 10 minutes of cognitive games/community-living skills training/relaxing scenery experiences, as appropriate.

**Table 1:** Some of the key considerations during program development to cater to the different conditions and disabilities of the users.

Element	Considerations
Hardware	Fully immersive simulation was adopted, with head-mounted display (HMD) devices and limb motion trackers used as the display method and control interface, respectively. Through the HMD, the images of virtual reality (VR) games were manifested in front of the users’ eyes. It enabled users with low vision to see the VR scenery clearly. Moreover, the HMD device was highly portable and could be used with minimal space requirement, which fitted well for a densely populated place such as Hong Kong. Button-free limb motion trackers allowed the users to participate in VR games by simple limb movements without relying on fine-motor control.
Virtual setting	An approximately 180-degree interaction zone within the VR environments was adopted, where interactive objects or training tasks were set at arm’s length or attached to users’ virtual hands. Hence, users could play the games safely in a sitting posture without moving or bending forward. Agile body and head movements were excluded or minimized from the gaming design to alleviate cybersickness such as dizziness and nausea. The backdrop and characters of VR games were not too fancy or stimulating to cater to users with limited cognitive and visual perceptions.
Game operation	Users only needed to move the button-free trackers with their limbs to a target position and hold for about 2 seconds. Then, a preprogrammed action would be triggered. Moreover, some of the VR games allowed users to either stand up or sit down to play, whereas some permitted the use of one or more limbs to play selectively (eg, left, right, or both), whichever suits the user, such that even those with mutilation could enjoy VR games. Motion-detection sensitivity was tailor made to the capability of users so that even the smallest movement of their limbs and the slowest reaction time could be detected. Then, those with minimal mobility could also easily participate in VR games using button-free trackers.
Game setting	Most of the games were designed with three levels of difficulty so that users and/or staff could choose the most suitable one according to their abilities. All games have timely and encouraging feedback and cues. By providing immediate feedback, users would realize that what they do in the real world could trigger something in the VR games. In addition to feedback, cues were provided in the form of images, words, and sounds, which served as guides to help users understand what they should do in the VR games. Staff could also use their own cues as additional guidance. Only positive reinforcement was adopted in the game design, allowing users to freely explore the VR games without any sanction and penalty (eg, score deduction). It also provided a sense of achievement to users and encouraged them to continuously engage in VR training.

**Table 2:** Virtual reality games in the training session.

Component	Virtual reality (VR) games
Physical	The games involved the upper limbs (*handball*), lower limbs (*football*), or full-body motions (*gatekeeping*). Users repeatedly mobilized their limbs and used their muscles, which aimed to help slow down the deterioration of bodily functions.
Cognitive (classification skills)	Nostalgic elements were employed to appeal to the users. Three signature games/scenes from a local amusement park that operated from the 1940s to the 1990s were included. The games, namely *Elephant Feeding*, *Feather Duster Throwing*, and *Coin Tossing*, were familiar to most Hong Kong older adults. They could provide not only cognitive stimulation but also a reminiscence of the old days. Another game, named *Home Items Locating,* simulated a home setting and required users to identify the appropriate items in the corresponding rooms.
Cognitive (reality orientation)	In the game named *Hong Kong Footprint*, users were virtually brought to familiar local places, including Shek Kip Mei, Man Mo Temple, Wong Tai Sin Temple, and Chun Yeung Street. Each scene was played with a soundtrack that briefly introduced the place. Along the VR journey, a trained staff member discussed the place that the user was watching. The experience allowed older adults to revisit familiar places and widened the horizons of residential care clients. These aimed to elicit pleasure and satisfaction, improve communication, and enhance self-esteem and social skills.
Community- living skills	The game *Seven Must-Dos Before Leaving Home* aimed to promote users’ awareness (especially older adults) of the seven safety measures that keep them and the living environment safe before leaving their homes. Another game named *MTR GO GO GO* aimed to help those with disabilities to develop skills in taking public transport and going to different places in Hong Kong by themselves. It simulated a Mass Transit Railway (MTR) station scene and aimed to familiarize users with travelling via the MTR under various conditions repeatedly and purposefully.
Relaxing scenery experiences	Four relaxing scenes, including *diving*, *river*, *starry sky*, and *grassland*, accompanied by relaxing music, were provided. The experience aimed to relieve users’ physical tension, calm their agitated mood, and engage with them in an interactive virtual environment. In the virtual scenes, various interactive objects were merged with a real 3D scene. Users could interact with them and trigger a particular preprogrammed effect. Additionally, the scenes aimed to provide dramatic multisensory stimulation for some frail users who could not engage in real sceneries that require limb motions.

### Procedure

Trained staff of the NGO identified and recruited potential participants to join the evaluation study. Upon recruitment, written informed consent was sought from the participants or their legal guardians. Eligibility screening and assessment were conducted by trained research assistants. Then, eligible participants engaged in 30-minute VR training three times a week for 6 consecutive weeks (giving a total of 18 sessions). Deviations were allowed according to the actual situation of the users. For example, if a week of training was missed due to sickness, the user was allowed to continue the program in the seventh week.

For each session, a trained staff member always accompanied the participant in the same room. The staff member was responsible to assist the user in putting on the head-mounted display (HMD) device and limb motion trackers (both hand and foot), to guide the user in playing the VR games, and to closely monitor the conditions of the user during game play to ensure safety. In between different training components, the participants could take off the HMD device and rest if needed. Any training component that was not applicable to the users would be skipped, for example, those with total loss of lower-limb function would not engage in lower-limb motion training. The participants could terminate the session whenever they wished. Adverse events were monitored and recorded as appropriate. After each session, the staff asked the participants for feedback on their experience of the VR games in that particular session.

### Data Collection and Outcome Measures

Demographic and medical information, which included age, gender, living arrangement (community-dwelling vs institutional setting), education level, dependence status, mobility status, cognitive status, intellectual disability status, visual impairment status, medical history (eg, stroke and fracture), and medical conditions (eg, mental illness and autism), were extracted from the records of the residents/members at baseline.

Health outcomes, including upper-limb dexterity, functional mobility, cognitive status, and happiness, were assessed by the trained research assistants at baseline (T0), 6 weeks after baseline (T1), and 3 months after baseline (T2). Participants with conditions that prohibited them from performing certain assessments were excluded from the analysis for that outcome.

Upper-limb dexterity was measured by the standard Box and Block Test (BBT). The BBT has been shown to be a valid measure of dexterity in the older population [22]. The participants were asked to transfer blocks from one compartment to the other in 1 minute according to a standard procedure [23]. The number of blocks transferred to the second compartment is counted, and a larger number indicates better dexterity.

Functional mobility was measured by the Timed Up and Go Test (TUG). The participants were asked to get up from an armchair, walk 3 meters, turn back, and return to a seated position according to a standard protocol [24]. The time required to finish the task at each round was recorded. Three measurements were made. Shorter walking time indicated better balance. The best (fastest) time of the three TUG trials was used [25].

Cognitive status was measured using the Montreal Cognitive Assessment 5-Minutes (Hong Kong Version) (HK-MoCA 5-Min) protocol. The validated assessment test covered the following four domains: attention, executive functions/language, orientation, and memory [26,27]. The total score ranged from 0 to 30, with a higher score indicating better cognitive status. The alternate version was not used in subsequent assessments, as literature suggested that the results were unlikely to be affected if the same assessment tool was used over the follow-up period [28]. If the participants were incapable of answering the HK-MoCA 5-Min, they were examined using the Benton Temporal Orientation Test (BTO), which involves only questions about orientation to time (including identifying the year, month, day, day of the week, and time of the day) [29]. An error score was calculated according to the Benton Temporal Orientation Scale (BTOS), with a lower error score indicating better cognitive status [30].

Happiness was measured using a single question “for most of the time, do you feel…” [31], accompanied with a 11-point Likert-scale ruler or 5/3/2 facial expression pictures about happiness [32]. Following the pretest protocol suggested for the Personal Wellbeing Index–Intellectual Disability (Chinese-Cantonese), the intellectual ability of respondents was assessed to determine the response options [32]. The options (for respondents with descending order of ability) included an 11-point scale (0-10), a 5-point scale (0-4), a 3-point scale (0-2), and a 2-point scale (0-1). Then, the score was standardized into units of “percentage of scale maximum,” with a possible range of 0 to 100 and higher scores indicating higher levels of happiness [32].

Usage time of VR training was collected via the built-in VR software system to ensure data accuracy. At the end of each session, the trained staff who accompanied the participants in the VR training collected qualitative feedback on the training experience, using the question “Do you like or dislike the VR training? Why?” The participants could provide multiple feedback responses, both positive and negative, if they wished. Any adverse health effects (such as dizziness) induced after playing the VR games were also recorded through self-reports and staff observations. The staff accompanying the participants in the VR training closely observed any discomfort experienced by the participants, in addition to explicitly asking the participants about any discomfort.

### Statistical Analysis

The primary outcomes were upper-limb dexterity and acceptance of VR training. The secondary outcomes were usage statistics, functional mobility, cognitive status, and happiness. SPSS version 25 (IBM Corp) was used for statistical analyses. A 5% significance level was adopted.

The participant characteristics were summarized using descriptive statistics. Characteristics of dropout participants were examined using a logistic regression model. VR training usage statistics in terms of the number of weeks engaged in VR training, number of total sessions of VR training attended, and duration of each VR training session were calculated and used to reflect the feasibility of the intervention. Linear mixed effect models were used to analyze the change in health outcomes, adjusting for age, gender, education level, living arrangement, disability, and medical history/condition. Assessment time was used as the independent variable, and its fitted coefficients indicated the temporal change in health outcomes in T1 and T2 as compared with T0. Missing data at follow-up were not imputed as the linear mixed effect models could cater for missing data. The standardized effect size in terms of the Cohen *d* index was calculated using the adjusted mean difference divided by the standard deviation [33]. Cohen *d* index values of 0.2, 0.5, and 0.8 indicated small, moderate, and large effect sizes, respectively.

Content analysis was performed for the qualitative feedback on the experience of VR training. First, the presence of concepts related to positive and/or negative comments was determined. Then, the presence of keywords and related concepts, such as tiredness, equipment, excitement, and exercise, was counted. Lastly, the frequency and percentage were calculated accordingly. The acceptance level of VR training experience was reflected by the number of participants providing positive comments, with a larger proportion indicating a higher level of acceptance. As the participants could provide multiple feedback responses, both positive and negative, when we calculated the proportion of participants using the total number of participants as the denominator, the proportion could exceed 100%. Therefore, in order to investigate factors associated with a higher level of acceptance, participants had to be further classified into the following three mutually exclusive groups: (1) only or mostly positive comments, (2) only or mostly negative comments, and (3) no comments or equal positive/negative comments. The proportions of participants in each group were calculated. Multiple multinomial regression was conducted to identify the associated factors. Adverse health events were also summarized.

### Ethics Approval

This study was approved by the Institutional Review Board of the University of Hong Kong/Hospital Authority Hong Kong West Cluster (reference number: UW19-336).

## Results

### Characteristics of the Participants

**Figure 1:**
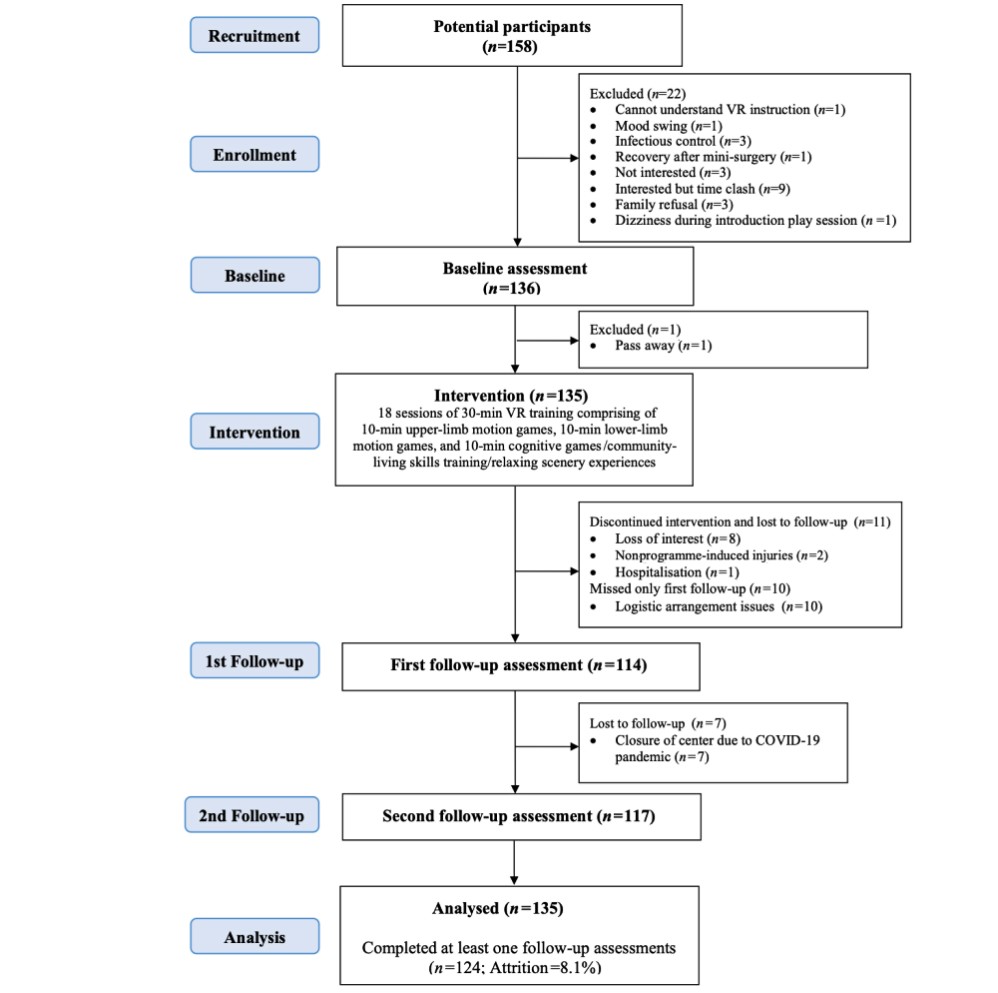
CONSORT (Consolidated Standards of Reporting Trials) flow diagram. VR: virtual reality.

A total of 158 participants were recruited from both institutional and community settings, including four homes for the severely disabled, eight homes for the intellectually disabled, six homes for the visually impaired, one old-age home, one community center for the intellectually disabled, and three elderly community centers. Of the 158 participants, 23 (14.6%) were not eligible, giving a total of 135 participants for the evaluation study (Figure 1). Table 3 summarizes the baseline characteristics of the 135 participants. The participants had a mean age of 62.7 years (SD 21.5). Additionally, 50.4% (68/135) of the participants were male, 76.3% (103/135) had an education level of primary school or below, or special educational needs, and 70.4% (95/135) were living in residential care settings. All participants had at least one type of disability, with 52.6% (71/135) having moderate-to-severe functional dependence, 78.5% (106/135) requiring walking aids or wheelchairs, 28.1% (38/135) having mild-to-severe cognitive impairment, 54.8% (74/135) having mild-to-severe intellectual disability, and 45.9% (62/135) having mild-to-severe visual impairment. Moreover, 9.6% (13/135) of the participants had a stroke history, 9.6% (13/135) had a fracture history, 13.3% (18/135) had mental illness, and 9.6% (13/135) had autism.

Additionally, 91.9% (124/135) of the eligible participants completed at least one follow-up. Some of them dropped out because of loss of interest (n=8), while a few of them did so because of nonprogram-induced injuries (n=2) and hospitalization (n=1). The logistic regression model showed that none of the participant characteristics was associated with dropout.

**Table 3:** Baseline characteristics of the participants (N=135).

Characteristic		Value, n (%) or mean (SD)
Mean age (years)		62.7 (21.5)
**Gender**		
	Male	68 (50.4)
	Female	67 (49.6)
**Educational level**		
	Primary or below, or special educational needs	103 (76.3)
	Secondary or tertiary	32 (23.7)
**Living arrangement**		
	Community dwelling	40 (29.6)
	Residential care setting	95 (70.4)
**Dependence level**		
	Independence or slightly dependence	64 (47.4)
	Moderate-to-severe dependence	71 (52.6)
**Mobility status**		
	Without auxiliary equipment	29 (21.5)
	Require walking aids	34 (25.2)
	Wheelchair bound	72 (53.3)
**Cognitive status**		
	No cognitive impairment	97 (71.9)
	Mild-to-severe cognitive impairment	38 (28.1)
**Intellectual disability status**		
	No intellectual disability	61 (45.2)
	Mild-to-severe intellectual disability	74 (54.8)
**Visual status**		
	No visual impairment	73 (54.1)
	Mild-to-severe visual impairment	62 (45.9)
**History of stroke**		
	No	122 (90.4)
	Yes	13 (9.6)
**History of fracture**		
	No	122 (90.4)
	Yes	13 (9.6)
**Mental illness**		
	No	117 (86.7)
	Yes	18 (13.3)
**Autism**		
	No	122 (90.4)
	Yes	13 (9.6)

### Usage Statistics of VR Training

A total of 1906 VR training sessions were conducted during the study period. Table 4 shows the summary of the usage statistics of VR training. Ninety-one individuals participated in the VR training for 6 weeks consecutively and another 15 participated for at least 6 weeks intermittently, giving a total of 106 participants (106/135, 78.5%) that met the suggested usage duration of 6 weeks. Over three quarters (103/135, 76.3%) of participants attended at least 13 sessions of VR training (ie, at least 70% of the proposed 18 sessions), and the majority (n=1382, 72.5%) of the 1906 sessions had a training time (excluding set-up time and rest time) of at least 20 minutes. The median total training time of the participants was 316.8 minutes. All of them participated in physical training games for a median of 275.0 minutes, and 116 of them participated in cognitive training (classification) games for a median of 33.8 minutes (Table 5). As for the difficulty level, 84.7% (1512/1786) of the *handball* game sessions, 88.7% (1295/1460) of the *football* game sessions, and 66.7% (909/1363) of the *classification* game sessions were at the easiest level (level 1) (Table 6). On the other hand, participants were able to play more advanced levels in *MTR GO GO GO*. Reasons for not complying with the usage protocol, including medical appointments or other engagements, limited resources, bad weather, technical problems, disability constraints, mood swings, sickness, and tiredness, were encountered in the scheduled sessions.

**Table 4:** Usage statistics of virtual reality training.

Usage statistics		Value, n (%)
**Number of weeks of VR^a^ training (n=135 participants)**		
	≥6 weeks consecutively	91 (67.4)
	≥6 weeks intermittently	15 (11.1)
	<6 weeks	29 (21.5)
**Number of sessions of VR training (n=135 participants)**		
	≥18 sessions	28 (20.7)
	13-17 sessions	75 (55.6)
	<13 sessions	32 (23.7)
**VR training time per session (n=1906 sessions)**		
	≥30 minutes	175 (9.2)
	20-29 minutes	1,207 (63.3)
	<20 minutes	524 (27.5)

^a^VR: virtual reality.

**Table 5:** Game time statistics according to game components.

Games	Number of participants	Time (minutes)					
		Mean	Median	Minimum	Maximum	SD	Range
All	135	289.3	316.8	6.0	595.0	129.6	6.0-595.0
Physical	135	244.0	275.0	4.0	390.0	108.6	4.0-390.0
Cognitive (classification)	116	43.0	33.8	2.0	177.8	35.0	2.0-177.8
Cognitive (reality orientation)	21	21.5	17.8	1.4	49.1	14.6	1.4-49.1
Community-living skills	18	16.8	15.1	3.3	42.2	12.4	3.3-42.2
Relaxing scenery experiences	27	13.7	10.5	2.6	50.0	12.1	2.6-49.9

**Table 6:** Difficulty levels of some virtual reality games played in the 1906 sessions.

Game	Sessions, n (%)			
	Level 1	Level 2	Level 3^a^	Mixed levels
Handball (n=1786 sessions)	1512 (84.7)	107 (6.0)	18 (1.0)	149 (8.3)
Football (n=1460 sessions)	1295 (88.7)	82 (5.6)	6 (0.4)	77 (5.3)
Classification (n=1363 sessions)	909 (66.7)	63 (4.6)	210 (15.4)	181 (13.3)
MTR GO GO GO (n=47 sessions)	9 (19.1)	22 (46.8)	15 (31.9)	1 (2.1)

^a^Including levels 3 and 4 in MTR GO GO GO.

### Health Outcomes

**Figure 2:**
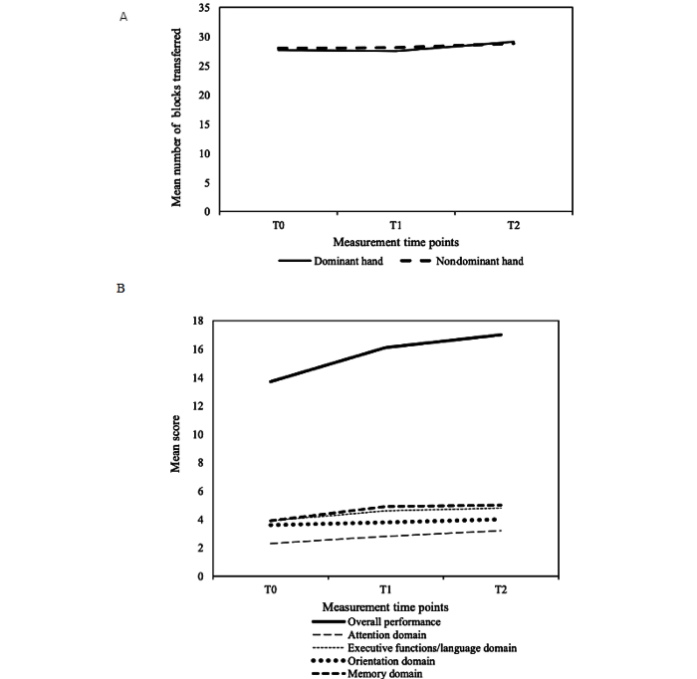
Change in health outcomes over three measurement time points (baseline [T0], 6 weeks after baseline [T1], and 3 months after baseline [T2]). (A) Block and Block Test (BBT), a larger number of blocks transferred indicates better upper-limb dexterity; *P* values for time difference: .008 (dominant hand) and .043 (nondominant hand). (B) Montreal Cognitive Assessment 5-Minutes (Hong Kong Version) (HK-MoCA 5-Min), a higher score indicates better cognitive status; *P* values for time difference: <.001 (overall performance), <.001 (attention domain), <.001 (executive functions/language domain), .004 (orientation domain), and <.001 (memory domain).

According to their conditions, the participants received varying health outcome assessments at baseline, with the BBT (n=134 for the dominant hand and n=128 for the nondominant hand), TUG (n=104), HK-MoCA 5-Min (n=94), BTO (n=30), and happiness scale (n=130) (Table 7). Among the health outcomes, significant improvement over time was noted in upper-limb dexterity (*P*=.008 for the dominant hand and *P*=.043 for the nondominant hand) and cognitive function (*P*<.001 for overall performance) (Figure 2), whereas there was no significant change in functional mobility (*P*=.14), orientation to time (*P*=.72), and happiness (*P*=.34).

**Table 7:** Baseline outcome measures of the participants (N=135).

Outcome measure		Number of participants	Value, mean (SD)
**BBT^a^ (in blocks)**			
	Dominant hand	134	27.7 (14.1)
	Nondominant hand	128	28.0 (14.7)
TUG^b^ (in seconds)		104	15.4 (11.8)
**HK-MoCA 5-Min^c^ score**			
	Overall performance	94	13.7 (7.8)
	Attention domain	94	2.3 (1.4)
	Executive functions/language domain	94	3.9 (2.3)
	Orientation domain	94	3.6 (2.1)
	Memory domain	94	3.9 (3.2)
BTO^d^ score (in error scores)		30	37.8 (25.9)
Happiness score		130	79.1 (30.0)

^a^BBT: Box and Block Test.

^b^TUG: Timed Up and Go Test.

^c^HK-MoCA 5-Min: Montreal Cognitive Assessment 5-Minute (Hong Kong Version).

^d^BTO: Benton Temporal Orientation Test.

Based on the linear mixed effect models (Table 8), the number of blocks transferred by the dominant hand significantly increased by 2.4 blocks (*P*=.007, Cohen *d*=0.17) and that by the non-dominant hand increased by 1.9 blocks (*P*=.04, Cohen *d*=0.13) when comparing T2 with T0. However, no significant differences in the number of blocks transferred were observed for both the dominant and nondominant hands when comparing T1 with T0.

In terms of the HK-MoCA 5-Min, the overall performance score significantly increased by 2.5 (*P*<.001, Cohen *d*=0.31) when comparing T1 with T0, and increased by 3.5 (*P*<.001, Cohen *d*=0.45) when comparing T2 with T0. Significant differences were also detected in T1 and T2 compared with T0 for all domains, except the orientation domain where an insignificant difference was reported for the comparison between T1 and T0. When comparing T2 with T0, the Cohen *d* of the domains ranged from 0.24 for orientation to 0.65 for attention.

**Table 8:** Change in health outcomes over the three assessment time points.

Outcome measures^a^			T1^b^ vs T0^c^							T2^d^ vs T0				
			Change^e^ (95% CI)		Effect size^f^		*P* value		Change^e^ (95% CI)			Effect size^f^		*P* value
**BBT^g^ (in blocks)**														
	Dominant hand	0.7 (−0.9 to 2.3)		0.05		.39		2.4 (0.9 to 4.0)			0.17		.002^h^	
	Nondominant hand	0.7 (−0.7 to 2.2)		0.05		.33		1.9 (0.4 to 3.3)			0.13		.01^h^	
TUG^i^ (in seconds)			−0.5 (−1.6 to 0.6)		−0.04		0.39		−1.1 (−2.2 to −0.007)			−0.09		.048^h^
**HK-MoCA 5-Min^j^ score**														
	Overall performance	2.5 (1.7 to 3.2)		0.31		<.001^h^		3.5 (2.8 to 4.3)			0.45		<.001^h^	
	Attention domain	0.5 (0.2 to 0.7)		0.33		.001^h^		0.9 (0.6 to 1.2)			0.65		<.001^h^	
	Executive functions/language domain	0.8 (0.4 to 1.1)		0.33		<.001^h^		1.0 (0.7 to 1.3)			0.43		<.001^h^	
	Orientation domain	0.3 (−0.005 to 0.6)		0.14		.054		0.5 (0.2 to 0.8)			0.24		.001^h^	
	Memory domain	1.0 (0.5 to 1.4)		0.30		<.001^h^		1.2 (0.7 to 1.6)			0.37		<.001^h^	
BTO^k^ score (in error scores)			2.6 (−7.4 to 12.6)		0.10		.61		−1.5 (−11.4 to 8.3)			−0.06		.76
Happiness score			−4.4 (−10.6 to 1.9)		−0.15		.17		−3.5 (−9.8 to 2.7)			−0.12		.26

^a^A better condition is represented by an increase in transferred blocks in the BBT, a decrease in time in the TUG, an increase in the HK-MoCA 5-Min score, a decrease in error scores in the BTO, and an increase in the happiness score.

^b^T0: baseline.

^c^T1: 6 weeks after baseline.

^d^T2: 3 months after baseline.

^e^The change in health outcomes over time were estimated using the linear mixed effect models, controlling for age, gender, education level, living arrangement, dependence level, mobility status, cognitive status, intellectual disability status, visual status, history of stroke, history of fracture, mental illness, and autism.

^f^Effect size is expressed in terms of the Cohen *d* index, where 0.2 indicates a small effect, 0.5 indicates a medium effect, and 0.8 indicates a large effect.

^g^BBT: Box and Block Test.

^h^Significant at <.05 level of significance.

^i^TUG: Timed Up and Go Test.

^j^HK-MoCA 5-Min: Montreal Cognitive Assessment 5-Minute (Hong Kong Version).

^k^BTO: Benton Temporal Orientation Test.

### Feedback From Participants

Among the 135 participants, 88 (65.2%) provided positive comments and 63 (46.7%) provided negative comments (multiple comments allowed), whereas 30 (22.2%) did not provide any comment. Among the 88 participants who provided positive comments, 53 (60.2%) reported the VR experience as innovative, interactive, fun, or exciting, 49 (55.7%) found the experience motivating, and 30 (34.1%) perceived the VR games as exercises. Meanwhile, among the 63 participants who provided negative comments, 26 (41.3%) complained about the VR equipment (such as the HMD device), 25 (39.7%) felt bored, 24 (38.1%) felt physically tired, 17 (27.0%) described the experience as scary, tense, or worrying, and 16 (25.4%) reported the usage as complicated. Moreover, 38 (60.3%) of those who indicated negative comments expressed such comments during the first two game sessions.

In terms of enjoyment, most participants enjoyed the *elephant feeding* game (n=11), followed by the *handball* game (n=5)*, football* game (n=5)*, diving scenery experience* (n=5), *feather duster throwing* game (n=3), *home items locating* game (n=3), and *coin tossing* game (n=1). At the same time, in terms of dislike, most participants disliked the *elephant feeding* game (n=11), followed by the *diving scenery experience* (n=3) and *seven must-dos before leaving home* game (n=1).

The participants were further classified into the following three groups: those who provided only or mostly positive comments (n=61, 45.2%), those who provided only or mostly negative comments (n=34, 25.2%), and those who had no comments or provided equal positive/negative comments (n=40, 29.6%). Based on multiple multinomial logistic regression, home-dwelling participants (*P*<.001) and those without autism (*P*=.008) had a much higher chance of providing positive comments. On the other hand, none of the participant characteristics was associated with a higher chance of expressing negative comments.

Apart from the general feedback, reports on mild discomfort were obtained in 25 of the 1906 (1.3%) game sessions (14 of 135 participants, 10.4%). The most common mild discomfort was dizziness (nine sessions from seven participants), followed by eyestrain (six sessions from three participants), hand/leg pain (three sessions from two participants), blurred vision (two sessions from one participant), eye redness (two sessions from one participant), hand tremors (two sessions from one participant), and cramps (one session from one participant). Over half (13/25, 52.0%) of these mild discomforts occurred in the first four sessions of the VR training. None of the participants reported severe discomfort. Moreover, staff reported technical problems in 63 of the 1906 (3.3%) game sessions.

## Discussion

### Overview

This evaluation study explored the feasibility and acceptability of a universal set of VR training games for users with different abilities and conditions. Additionally, the improvement in health outcomes over time was assessed. The findings from this study can inform the future development and application of VR training to heterogeneous users in different settings.

### Feasibility of Providing VR Training to Users With Different Disabilities

Few VR games have been designed for the broad coverage of different populations. Our experience revealed the possibility that people with different disabilities could enjoy the same set of VR games. With different training components, clients with different disabilities could participate in suitable types of training. Nevertheless, manpower resources had to be invested in addition to the VR system, since close supervision of the users by trained staff was required.

With a rehabilitation goal, we proposed a training protocol of 3 days a week for 6 weeks, with a target of 18 sessions. Over 76% of the participants attended 13 or more sessions. Adherence to the proposed number of training sessions was comparable to that reported in the literature [34]. Flexibility for makeup training was allowed if participants were not able to attend certain sessions. Usage statistics showed that the majority of sessions could offer at least 20 minutes of game time. However, the game time for cognitive training/community-living skills training/relaxing scenery experiences was substantially less than that for physical training. One explanation could be the limit of the session time. These components were placed as the last training components of each session, and thus, they might have been terminated if time was running out. Scheduling was a critical element to the successful implementation of the program. To facilitate a 30-minute training time, the manpower, equipment, and venue had to be made available for at least 45 minutes to allow for the necessary time for setup, instructions, and breaks. Moreover, as reality orientation, community-living skills training, and relaxing scenery experiences were available for use at a later stage, usage statistics were substantially less than those for the classification game. Despite the users’ inability to attend all 18 sessions or spend 30 minutes of training time per session, there was a significant improvement in cognitive function and upper-limb dexterity. Hence, we propose to retain the current usage protocol until a new one with reduced usage is tested.

In our experience, mild discomfort was reported by 14 of 135 participants (10.4%). In a local study examining the effects of VR cognitive stimulation activity among community-dwelling older adults (n=236), 1.4% of them reported severe discomfort regarding fatigue and eye strain, blurred vision, and dizziness after 20 to 25 minutes of VR exposure [17]. However, mild discomfort was not reported. In another study conducted by US researchers, it was reported that 12% of older people with Alzheimer disease/mild cognitive impairment and 19% of older people without such conditions dropped out from the VR game because of simulation sickness [35]. The proportion of participants reporting adverse outcomes among our users was comparable, if not lower, than the proportions reported in the literature. Therefore, participants with disabilities would not put themselves at a higher risk of adverse events. At the same time, some participants reported physical tiredness after VR training. While this study did not quantitatively investigate tiredness as an outcome, a meta-analysis suggested an insignificant difference in terms of the presence of tiredness after VR training as compared with participants engaging in traditional exercises [36].

### Acceptance of VR Training Among Users at an Advanced Age or With Various Disabilities

Generally, most of the participants accepted the use of the VR training, as 65.2% of the participants indicated positive comments. Those living in the community and those without autism tended to provide more positive remarks. This might be because these participants were more expressive of themselves. On the other hand, the HMD device and limb motion trackers might have induced discomfort, which was the major reason for negative comments. To enhance their experience and satisfaction in terms of fit and comfort, hardware designers may develop lighter devices. To accommodate the special needs of clients with limitations in head and neck movements, sensors should also be further adjusted so that clients do not need to maintain an upright position throughout the sessions.

Some participants also perceived the VR games as complicated, but they could still complete the session under the supervision of trained staff. Although some users claimed the VR training was boring, there was no evidence showing that such negative remarks were related to the proposed VR training for 18 sessions. Indeed, those who made negative comments had such perceptions in the first two sessions. This might suggest the need for more briefings or orientations prior to the use of VR training. In the current program, the scoring regime had been modified to increase the users’ sense of achievement. However, there was no personalized scoring regime to differentiate game difficulty levels for different disabilities over time. In future development, such features should be incorporated for motivating clients to continue training and promoting a higher sense of achievement where progression could be felt by users.

The VR games that involved physical activity training were well received by users. It was interesting to learn that the VR cognitive-training game featuring the elephant kept in a zoo in Hong Kong in the 70s received both favorable and unfavorable reviews from the users. Some loved it probably because it recalled memories of the old days, while some disliked it probably because they were scared by the elephant’s trumpet, which was close to that in real life. Future design of VR scenery should take a balanced approach among excitement, calmness, and boredom.

### Efficacy of VR Training in Improving the Health Outcomes of Participants

Using a single-arm pretest-posttest design, the evaluation study showed significant improvement in upper-limb dexterity assessed by the BBT and cognitive function assessed by the HK-MoCA 5-Min. According to the literature, an increase of 2 points in the MoCA full version score was generally considered as a minimal clinically important difference (MCID) for older adults or stroke survivors, which was about 0.5 standard deviations as defined by the distribution-based approach [36-38]. However, the MCID in terms of the MoCA 5-Min version has not been discussed in the literature. Hence, we used a distribution-based approach of 0.5 standard deviations, and considered a Cohen *d* index of 0.5 as the MCID. In our evaluation study, the HK-MoCA 5-Min overall score increased by 3.5 units as compared with baseline. In terms of the standardized effect size, the HK-MoCA 5-Min score had a Cohen *d* index of 0.45, which aligned with this reference. As understanding and following instructions to play VR games involve cognitive processing, the improvement in cognitive function might be enhanced by playing all the games and not necessarily restricting playing to the sessions involving cognitive VR games.

As for the BBT, there was no well-established MCID. Literature suggested that a difference of 6 to 8 blocks is considered as the smallest real difference beyond measurement error among stroke survivors [39], which would translate to a Cohen *d* index of 0.4 to 0.6 based on the standard deviation estimated in our study. Since the effect size of the BBT achieved in our study was only 0.13 to 0.17, we had to be cautious in interpreting the clinical significance of such a change. For functional mobility, although there was a favorable trend in improvement, such improvement did not reach statistical significance. It appeared that training intensity, particularly when participants took a sitting position when playing the games, was not sufficient to achieve improvement in functional mobility.

Our results are consistent with recent reviews, which found that VR training attained small-to-moderate effects in terms of physical performance [40] and cognitive function [41]. Despite the significant improvement in the oriental domain of the HK-MoCA 5-Min scale, there was an insignificant change in the BTO score. Further research is suggested to examine the reasons. Nevertheless, we would be cautious about clinical significance in terms of the improvement in the BBT. Our insignificant results in terms of mobility are also consistent with the absence of an effect in terms of gait as reported by a review [40].

The evaluation study did not show any significant change in the happiness level. This is consistent with a recent systematic review of VR exercise training that reported insignificant effects on psychological outcomes such as calmness and enjoyment [40]. The review also suggested that immersive components of VR training and longer follow-up periods tended to be associated with smaller benefits, but statistical significance was not found. As VR games are designed to promote functional and cognitive ability, if happiness is to be enhanced, the content of VR games might need to be adjusted and a regular playing schedule might not need to be applied.

A supplementary analysis testing the interactions between different subgroups (dependence level, mobility status, cognitive status, intellectual disability status, visual status, stroke history, fracture history, mental illness, and autism) and time points was performed using linear mixed effect models. Insignificant interaction terms were found for all those explored, except a few, which might have occurred by chance. There was insufficient evidence for the difference in the change of outcomes between different subgroups, and future studies could revisit this with a more vigorous design.

### Strengths and Limitations

The strengths of this evaluation study were its large sample size, adoption of validated scales as outcome measures, and long follow-up period. Qualitative feedback from the participants also informed the strengths and weaknesses of the VR training program. However, the study has some limitations. First, the lack of a control group might have limited the interpretation of the results that only efficacy instead of effectiveness could be investigated. Second, people with hearing impairment, those who were bed bound, and those with disability in turnaround were not included in the study, as the current system could not cater to their special needs, and the potential benefits or adverse effects in these users are unclear from this study. The uses of various cognitive training components, community-living skills training, and scenery experiences were not standardized, and some games were not available at the beginning of the program, resulting in vast variations in game time for the components. In addition, progression of the game difficulty level was not standardized in the usage protocol, and this should be considered in future studies. As qualitative feedback responses were collected at the end of each game session, investigation by game components could not be performed. A future study should continue to investigate the optimal modalities in the domains of physical, cognitive, and psychological training for people with different vulnerable health conditions, and a controlled trial would be preferred.

### Conclusions

A set of VR training games for rehabilitation could be applied to individuals with heterogeneous abilities. Our VR games were acceptable to local older adults and those with different disabilities. Benefits in upper-limb dexterity and cognitive function were observed despite partial compliance to the training protocol. Service providers could refer to our experiences when developing VR training systems for their clients.

## Abbreviations

BBT: Box and Block Test
BTO: Benton Temporal Orientation Test
BTOS: Benton Temporal Orientation Scale
HK-MoCA 5-Min: Montreal Cognitive Assessment 5-Minutes (Hong Kong Version)
HMD: head-mounted display
MCID: minimal clinically important difference
NGO: nongovernment organizations
TUG: Timed Up and Go Test
VR: virtual reality
